# Targeting Bruton’s Tyrosine Kinase in Inflammatory and Autoimmune Pathologies

**DOI:** 10.3389/fcell.2021.668131

**Published:** 2021-06-04

**Authors:** Stefan F. H. Neys, Rudi W. Hendriks, Odilia B. J. Corneth

**Affiliations:** Department of Pulmonary Medicine, Erasmus MC, University Medical Center, Rotterdam, Netherlands

**Keywords:** Bruton’s tyrosine kinase (BTK), B cells, myeloid cells, inflammation, autoimmunity, small-molecule inhibitor

## Abstract

Bruton’s tyrosine kinase (BTK) was discovered due to its importance in B cell development, and it has a critical role in signal transduction downstream of the B cell receptor (BCR). Targeting of BTK with small molecule inhibitors has proven to be efficacious in several B cell malignancies. Interestingly, recent studies reveal increased BTK protein expression in circulating resting B cells of patients with systemic autoimmune disease (AID) compared with healthy controls. Moreover, BTK phosphorylation following BCR stimulation *in vitro* was enhanced. In addition to its role in BCR signaling, BTK is involved in many other pathways, including pattern recognition, Fc, and chemokine receptor signaling in B cells and myeloid cells. This broad involvement in several immunological pathways provides a rationale for the targeting of BTK in the context of inflammatory and systemic AID. Accordingly, numerous *in vitro* and *in vivo* preclinical studies support the potential of BTK targeting in these conditions. Efficacy of BTK inhibitors in various inflammatory and AID has been demonstrated or is currently evaluated in clinical trials. In addition, very recent reports suggest that BTK inhibition may be effective as immunosuppressive therapy to diminish pulmonary hyperinflammation in coronavirus disease 2019 (COVID-19). Here, we review BTK’s function in key signaling pathways in B cells and myeloid cells. Further, we discuss recent advances in targeting BTK in inflammatory and autoimmune pathologies.

## Introduction

Loss of immunological tolerance associated with the activation of autoreactive B cells and their differentiation into autoantibody-producing cells are important pathogenic features in human systemic autoimmune disease (AID). B cell receptor (BCR) signaling is crucial for B cell activation, survival and differentiation and, therefore, reflects a potential therapeutic target for AID. Bruton’s tyrosine kinase (BTK) is a cytoplasmic protein belonging to the family of TEC (tyrosine kinase expressed in hepatocellular carcinoma) kinases. BTK is renowned for its critical role in BCR signaling and was originally identified as the gene defective in X-linked agammaglobulinemia (XLA) patients (Tsukada et al., 1993; Vetrie et al., 1993). Based on the therapeutic benefit of the anti-CD20 antibody rituximab to deplete mature B cells in AID, strategies were developed to discover selective BTK inhibitors (BTKi) for the treatment of rheumatoid arthritis (RA; Pan et al., 2007). These BTKi were designed to covalently and irreversibly bind BTK at the cysteine 481 residue in the catalytic domain. Interestingly, the high potential of these inhibitors, such as ibrutinib and acalabrutinib, to modulate BCR signaling led to their rapid implementation in the treatment of several B cell malignancies (Hendriks et al., 2014).

Soon after the discovery of its crucial function in BCR signaling, the involvement of BTK in several other signaling routes in B cells and myeloid cells was demonstrated (Table 1). This fueled research into the effects of BTKi in the context of inflammatory and AID by solely targeting BCR signaling or by targeting multiple pathways in several cell types simultaneously. The potential use of BTKi for the treatment of AID is currently being explored *in vitro*, and *in vivo* in animal models and clinical trials. In this review, we summarize BTK’s function in key signaling pathways in B cells and myeloid cells, and we discuss recent advances in targeting BTK in inflammatory and autoimmune pathologies.

**TABLE 1:** The role of BTK in signaling pathways in various cell types.

**Cell type**	**Via**	**Process in which BTK plays a role**	**References**
B cell	BCR; TLR	Cytokine production (IL-6, TNFα, IFNγ, IL-10, IL-12)	Halcomb et al., 2008; Lee et al., 2008a; Corneth et al., 2016; von Borstel et al., 2019; Torke et al., 2020
	BCR; BAFFR; TLR	Proliferation, differentiation & immunoglobulin production	Wicker and Scher, 1986; Khan et al., 1995; Di Paolo et al., 2011; Haselmayer et al., 2019; von Borstel et al., 2019
	BCR	Antigen presentation (via MHC-II) and co-stimulation (via CD86 and CD69)	Kenny et al., 2013; Haselmayer et al., 2019
	BCR	Integrin-mediated adhesion of B cells to VCAM-1 and fibronectin	Spaargaren et al., 2003; de Rooij et al., 2012
	BAFFR	Homeostatic B cell survival	Shinners et al., 2007; Schweighoffer et al., 2013
	CXCR4; CXCR5; CCR7	Chemotaxis and homing to and within lymphoid organs	de Gorter et al., 2007; de Rooij et al., 2012
	IL-5R	Proliferation and differentiation	Hitoshi et al., 1993; Sato et al., 1994; Koike et al., 1995
Conventional DC	TLR7; TLR9	IFN-β production	Li et al., 2014
	TLR4	DC maturation and cytokine production	Kawakami et al., 2006; Natarajan et al., 2016
Plasmacytoid DC	TLR9	Cytokine production (IFNα, TNFα and IL-6) and expression of CD40, CD86, and CD69	Wang J. et al., 2014
Mast cell	FcεRI	Degranulation and cytokine production (IL-2, IL-3, IL-4, TNFα, IL-6)	Hata et al., 1998; Chang et al., 2011; Iyer et al., 2011; Soucek et al., 2011
	FcγR	Degranulation and cytokine production (TNFα, IL-8, MCP-1)	Chang et al., 2011
Neutrophil	GM-CSFR/TLR	Maturation and function	Fiedler et al., 2011
	FcγR/TLR4	Degranulation, oxidative burst, pathogen engulfment & cytokine production	Prezzo et al., 2019; Blez et al., 2020
	Fpr-1	fMLP-driven Mac-1-activation and infiltration into inflamed tissue	Gilbert et al., 2003; Volmering et al., 2016; Herter et al., 2018
	PSGL-1	E-selectin triggered activation of β_2_-integrin	Mueller et al., 2010; Yago et al., 2010
	NLRP3	Inflammasome activation and thereby IL-1β secretion	Ito et al., 2015; Liu et al., 2017
	TREM-1	Degranulation, oxidative burst and L-selectin shedding	Stadler et al., 2017
Basophil	FcεRI	Degranulation and cytokine production	Kneidinger et al., 2008; MacGlashan et al., 2011; Smiljkovic et al., 2017; Haselmayer et al., 2019
Monocyte	FcγR	Cytokine production (TNFα, IL-6, MCP-1, IL-1β)	Chang et al., 2011; Di Paolo et al., 2011; Ren et al., 2016
Macrophage	TLR2/4	Microbicidal activity (via nitric oxide (NO) production), cytokine production (TNFα, IL-1β) and M1 polarization	Mukhopadhyay et al., 1999, 2002; Mangla et al., 2004; Ni Gabhann et al., 2014; de Porto et al., 2019
	FcγR	Cytokine production (TNFα, IL-6, IL-1β, MCP-1)	Chang et al., 2011; Di Paolo et al., 2011; Hartkamp et al., 2015
	CD40	Cytokine (IL-6, IL-8, TNFα, IL-10) and NO production	Mukhopadhyay et al., 1999; Hartkamp et al., 2015
	DDX41	IFN-type I response	Lee et al., 2015
	NLRP3	Inflammasome activation and thereby IL-1β secretion	Ito et al., 2015; Liu et al., 2017
	M-CSFR	Survival	Melcher et al., 2008
Osteoclast	RANK	Maturation and differentiation	Lee et al., 2008b; Shinohara et al., 2008

## BTK Signaling Pathways

### Prosurvival Signaling in B Cells

B cell development takes place in the bone marrow. It is characterized by the ordered rearrangement of immunoglobulin heavy and light chain gene segments, leading to expression of a unique BCR. The random nature of this V(D)J recombination process inevitably generates BCRs that recognize self-antigen. However, multiple checkpoints ensure counterselection of these autoreactive B cells during development based on BCR specificity (Wardemann et al., 2003). These checkpoints are critical because autoreactive B cells, when activated, can have multiple pathogenic functions. These include the production of autoantibodies and pro-inflammatory cytokines, stimulation of tertiary lymphoid organ formation and antigen presentation to autoreactive T cells.

The survival of circulating B cells requires signals from both BCR and B cell activating factor (BAFF) receptor (BAFFR; Lam et al., 1997; Sasaki et al., 2004). This BCR prosurvival signaling is antigen-independent and is referred to as “tonic” signaling. It differs from stronger signals induced by cognate antigen binding, leading to activation and proliferation of B cells. BAFF-transgenic mice and mice with B cell-specific BTK overexpression (CD19-hBtk) develop autoimmune pathology resembling human systemic lupus erythematosus (SLE) and primary Sjögren’s syndrome (pSS; Thien et al., 2004; Kil et al., 2012).

**FIGURE 1:**
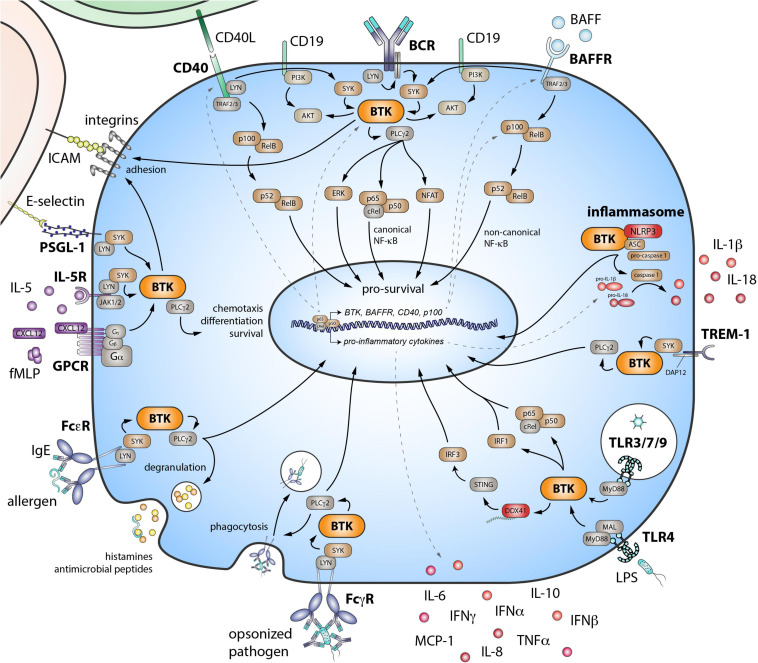
Role of Bruton’s tyrosine kinase (BTK) in various signaling pathways in B cells and myeloid cells. BTK is critical downstream of the B cell receptor (BCR). The BAFF receptor (BAFFR) transduces signals by coopting the BCR. The co-stimulatory receptor CD40 also signals via both the noncanonical NF-κB pathway and BTK. Eventually, these signaling pathways lead to the activation of downstream transcription factors—important for survival, differentiation, proliferation, and cytokine production of B cells. BTK also functions in inflammasome activation and in signaling downstream pattern recognition receptors, including triggering receptor expressed on myeloid cells 1 (TREM-1) and the Toll-like receptor (TLR) family. Activating FcγRs via BTK signaling, can stimulate cells to initiate cytokine production, phagocytosis, and microbicidal activity of engulfed pathogens. FcεRs can bind IgE and are mostly expressed on mast cells and basophils. When cross-linked, these receptors also signal via BTK, resulting in the quick release of histamines and antimicrobial peptides via degranulation. BTK is also involved in downstream signaling of G-protein coupled receptors (GPCR), such as chemokine and cytokine receptors. E-selectin–driven engagement of PSGL-1 induces downstream signaling via BTK to activate integrins. SYK, spleen tyrosine kinase; PLCγ2, phospholipase Cγ2; PI3K, phosphoinositide 3-kinase; ERK, extracellular signal-regulated kinase; NF-κB, nuclear factor-κB; TRAF, tumor necrosis factor receptor-associated factor; BAFF, B cell activating factor of the tumor necrosis factor superfamily; NLRP3, NLR family pyrin domain containing 3; ASC, apoptosis-associated speck like protein containing a caspase recruitment domain; DAP12, DNAX activation protein of 12 kDa; MyD88, myeloid differentiation primary response 88; MAL, MyD88 adaptor-like; IRF, interferon regulatory factor; DDX41, DEAD-box helicase 41; STING, stimulator of interferon genes; IL, interleukin; IFN, interferon; MCP-1, monocyte chemoattractant protein-1; TNFα, tumor necrosis factor α; FcγR, Fcγ receptor; FcεR, Fcε receptor; fMLP, N-Formylmethionyl-leucyl-phenylalanine; CXCL12, C-X-C-motif chemokine ligand 12; JAK, Janus kinase; PSGL-1, P-selectin glycoprotein ligand-1; ICAM, intercellular adhesion molecule.

B cell receptor engagement initiates intracellular signaling that leads to the phosphorylation of spleen tyrosine kinase (SYK; Figure 1). Subsequent recruitment of BTK to the cell membrane enables SYK to activate BTK through phosphorylation at Y551, followed by BTK autophosphorylation at Y223 (Rawlings et al., 1996). Active BTK can then phosphorylate phospholipase Cγ2 (PLCγ2). The formation of this BCR signalosome generates a Ca^2+^ influx and leads to activation of multiple downstream signaling pathways and transcription factors, including nuclear factor of activated T cells (NFAT), extracellular signal-regulated kinase (ERK), and nuclear factor (NF)-κB. These are crucial for B cell survival, proliferation, and differentiation. BTK has a central role in the BCR signaling pathway given the phenotype of XLA patients and the finding that BTK inhibition leads to a block in downstream signaling (Honigberg et al., 2010).

B cell activating factor is a ligand for three receptors: transmembrane activator and CAML interactor (TACI), B cell maturation antigen (BCMA), and BAFFR. The latter is most important for survival of mature naïve B cells by activating the noncanonical NF-κB pathway. Studies show that the BAFFR also transduces its signals by crosstalk with the BCR, involving SYK and BTK, leading to canonical NF-κB signaling (Figure 1; Shinners et al., 2007; Schweighoffer et al., 2013).

An important co-stimulatory receptor for T cell dependent B cell responses is CD40. Its ligand CD154 (CD40L) is expressed by activated T cells and, next to homeostatic proliferation of the naïve B cell pool, supports B cell differentiation and maturation (Schwartz et al., 2014). Signaling downstream of CD40 activates the noncanonical NF-κB pathway (Figure 1). However, BTK is also activated (Brunner et al., 2002), and the expression of BCR signaling proteins—including BTK—is enhanced (Kil et al., 2012). Interestingly, BAFF stimulates CD40 expression and CD40L co-stimulation from T cells in a BAFFR-dependent way (Zhang et al., 2016).

Therefore, BCR/BAFFR/CD40 signaling acts in a self-amplifying loop, both directly as prosurvival signals and indirectly by enhancing transcription of these prosurvival receptors and their downstream signaling molecules (Smith and Cancro, 2003; Stadanlick et al., 2008; Yu et al., 2008; Castro et al., 2009; Smulski and Eibel, 2018). It is hypothesized that, in AID, a disbalance in survival signals causes escape of autoreactive B cells from negative checkpoints. Therefore, modulating these B cell signaling pathways simultaneously via BTKi offers potential in targeting pathogenic B cells and constraining overt B cell activation.

### BTK Function Beyond B Cell Signaling

#### Pattern Recognition Receptors

Bruton’s tyrosine kinase is also implicated in signaling of various pattern recognition receptors (PRRs), a group of germline-encoded sensors expressed by innate and adaptive immune cells. PRRs have a pivotal role in sensing pathogen- and damage-associated molecular patterns and are expressed on the cell surface, intracellularly within endosomes or in the cytoplasm. BTK is involved in toll-like receptor (TLR) signaling, in which it interacts with their intracellular signaling domain and with the downstream adaptor molecules MyD88 (myeloid differentiation primary response 88) and MAL (MyD88-adaptor-like) (Hendriks et al., 2014). Similar to the BAFFR, TLR4 is thought to transduce signals through the BCR (Schweighoffer et al., 2017). BTK interacts directly with the cytoplasmic sensor NLR family pyrin domain containing 3 (NLRP3) and its adaptor ASC (apoptosis-associated speck like protein containing a caspase recruitment domain) and is, thus, involved in inflammasome and caspase-1 activation and subsequent interleukin (IL)-1β and IL-18 production (Ito et al., 2015). Engagement of triggering receptor expressed on myeloid cells-1 (TREM-1) induces BTK activation and leads to inflammatory responses (Ormsby et al., 2011). Last, BTK phosphorylates DEAD-box helicase 41 (DDX41) and stimulates its binding to dsDNA and activation of the STING (stimulator of interferon genes) pathway (Lee et al., 2015). All these pathways lead to downstream signaling and activation of transcription factors, such as activator protein 1 (AP-1), NF-κB, and/or interferon regulatory factors (IRFs), promoting differentiation, survival, and pro-inflammatory cytokine production (Figure 1; Weber et al., 2017).

Because BTK functions in both TLR and BCR signaling, B cell activation with TLR ligands can lead to synergistic effects when combined with BCR stimulation (Kenny et al., 2013). TLR signaling via NF-κB enhances expression of BTK and BAFFR (Kil et al., 2012; Abu-Rish et al., 2013). Hence, TLR triggering adds to this self-amplifying loop of multiple stimuli (BCR/CD40/BAFF) that support B cell survival. A disbalance in these signals can result in a disturbed survival of pathogenic B cells. Therefore, BTKi may show potential in the treatment of inflammatory and AID by inhibitory effects on PRR signaling in both B and myeloid cells.

#### Fc, Cytokine, and Chemokine Receptors and Integrin Activation

Antibodies exert their function—pathogen neutralization and opsonization—by complement activation and via Fc receptor (FcR) signaling involving BTK (Chang et al., 2011; Fiorcari et al., 2016; Figure 1). Activation of FcRs on myeloid cells induces release of antimicrobial factors via degranulation, and it stimulates de novo cytokine production, phagocytosis, and antigen presentation. Although these tools are crucial in the clearing of pathogens, these can be pathogenic in AID. Last, BTK also functions in downstream signaling of chemokine receptors (de Gorter et al., 2007), cytokine receptors (Sato et al., 1994; Matsuda et al., 1995) and in integrin activation (Spaargaren et al., 2003; Yago et al., 2010; Volmering et al., 2016; Herter et al., 2018). Thus, for both B and myeloid cells, BTK is important in processes controlling cell localization, survival, and adhesion and migration into site of inflammation.

## BTK and BTKi in B Cell–Mediated Autoimmune Disease

Following the production of first-line BTKi, such as ibrutinib, second-generation (mostly covalent) inhibitors with higher specificity, lower side effects, and their own unique properties were developed (Estupiñán et al., 2021; von Hundelshausen and Siess, 2021). Next to these small-molecule inhibitors, other types of inhibitors are being investigated, such as small-interfering RNAs targeting BTK production (Zhao et al., 2019). In this next section, we discuss the most important and most recent advances in the targeting of BTK in the context of inflammatory and AID.

### Rheumatoid Arthritis

Bruton’s tyrosine kinase-deficiency in mice is protective in several experimental autoimmune arthritis models (Jansson and Holmdahl, 1993; Nyhoff et al., 2016). Protection appeared to be largely attributable to its role in B cells although BTK may also contribute to disease through macrophages (Horwood et al., 2006; Ni Gabhann et al., 2014) and osteoclasts/osteoblasts (Hayer et al., 2008; Shinohara et al., 2008). This is also evident from animal studies in which BTKi ameliorated B cell–dependent but also B cell–independent myeloid-mediated arthritis (Chang et al., 2011; Di Paolo et al., 2011; Caldwell et al., 2019; Haselmayer et al., 2019; Angst et al., 2020; Liu et al., 2021).

In human RA, dysregulated BCR signaling may lead to aberrant B cell activation and loss of tolerance. BTK protein and phosphorylation (pBTK) were increased in peripheral blood B cells of anti-citrullinated protein antibody positive RA patients (Corneth et al., 2017). These protein levels correlated with pathogenic T cell subsets and pBTK expression correlated with rheumatoid factor levels in circulation (Wang et al., 2015). Furthermore, RA synovial tissue cultured with BTKi showed decreased pro-inflammatory cytokine production (Hartkamp et al., 2015). Together, these data suggest BTKi may be a beneficial therapeutic option in RA. Indeed, fenebrutinib showed efficacy at higher doses, comparable to tumor necrosis factor (TNF)α-inhibitor adalimumab, and reduced pro-inflammatory cytokine and autoantibody levels (Cohen et al., 2020). However, several other studies with BTKi showed only mild effects on disease severity, including spebrutinib (Schafer et al., 2020), evobrutinib (NCT02784106, NCT03233230), and tirabrutinib (NCT02626026).

### Primary Sjögren’s Syndrome

In an IL-14α-driven mouse model of pSS, BTK-deficiency did not protect against disease development (Shen et al., 2016). However, CD19-hBtk mice develop a spontaneous pSS/SLE-like autoimmune phenotype, including lymphocytic infiltrates in the salivary glands (Kil et al., 2012). This phenotype is dependent on B–T cell interaction (Corneth et al., 2016). Similarly, a subset of patients with active pSS had increased BTK protein and pBTK levels in circulating B cell subsets, which correlated with numbers of infiltrating T cells in the parotid gland and normalized following abatacept treatment (Corneth et al., 2017). Interestingly, enhanced BTK expression was already present in transitional and naïve B cells, which had a more activated phenotype and showed loss of tolerance in pSS (Corneth et al., 2017; Glauzy et al., 2017). Integrated BCR, TLR, and TACI signaling can induce autoantibody production by transitional B cells (Du et al., 2018). B cell–depleting studies in pSS have yielded contradicting results, possibly due to persistence of pathogenic B cells in the salivary glands, linked to high BAFF levels (Hamza et al., 2012; Cornec et al., 2016; Dorner et al., 2019). Importantly, BTK overexpression may be associated with increased risk of lymphoma development in pSS (Duret et al., 2019). BTKi may, therefore, be an interesting therapeutic strategy in pSS. Currently, a phase II clinical trial with remibrutinib in pSS is recruiting (NCT04035668).

### Systemic Lupus Erythematosus

Bruton’s tyrosine kinase-deficient mice and BTKi-treated mice are protected in a wide range of experimental models of systemic SLE (Rip et al., 2018). Efficacy is attributed not only to inhibition of BCR signaling—thereby reducing autoantibody levels—but also to TLR and FcR signaling in monocytes and macrophages, important drivers of renal damage in SLE. BAFF transgenic and CD19-hBTK mice develop a spontaneous pSS/SLE-like phenotype, featuring antinuclear antibodies and immunoglobulin deposition in the kidneys (Mackay et al., 1999; Kil et al., 2012). In SLE patients, increased BTK expression in peripheral B cells was linked to lupus nephritis and correlated with disease severity (Kong et al., 2018). In a mouse model of lupus nephritis, BTKi lead to remission (Chalmers et al., 2017). BTKi in SLE are currently being tested in several clinical trials (NCT02537028, NCT04305197, NCT03878303, and NCT02829541). A phase II clinical trial with the noncovalent BTKi fenebrutinib did not meet its primary end point (Isenberg et al., 2019) although strong immunomodulatory effects were shown. More studies are required to ascertain efficacy of BTKi in SLE.

### Systemic Sclerosis

Systemic sclerosis (SSc) is a very heterogeneous disease of unknown etiology. However, as >90% of patients harbor autoantibodies, B cells are thought to play a major role in SSc (Sakkas and Bogdanos, 2016). Genetic susceptibility studies implicate BCR signaling in disease pathogenesis (Dieude et al., 2009; Gourh et al., 2010; Rueda et al., 2010). Circulating BAFF levels were increased in SSc patients (Matsushita et al., 2007) and BAFF blockade modulated scleroderma phenotype in a bleomycin-mediated mouse model (Matsushita et al., 2018). *In vitro* treatment of SSc B cells with ibrutinib reduced IL-6, TNFα, and SSc-specific autoantibody production following TLR stimulation (Einhaus et al., 2020). Though further research is needed, these results indicate BTKi may be a therapeutic option in SSc.

### Multiple Sclerosis

Multiple sclerosis (MS) is a demyelinating AID of the central nervous system (CNS). B cells are thought to play an important role in MS pathogenesis as shown by the clinical success of rituximab treatment (Kinzel and Weber, 2016). In experimental autoimmune encephalitis, a mouse model for MS, BTKi ameliorated disease (Torke et al., 2020). Compared with other AID and healthy controls, MS B cells did not show increased BTK protein expression or pBTK levels upon BCR stimulation (Torke et al., 2020). A phase II clinical trial with evobrutinib showed promising clinical results at the highest dose (Montalban et al., 2019). Trials with tolebrutinib in relapsing and progressive forms of MS are currently running (NCT04410978, NCT04410991, and NCT04458051). As BTKi are small-molecule inhibitors, they may be better suited in entering the CNS and reaching pathogenic B cells than therapeutic antibodies such as rituximab (Dolgin, 2021).

### Type I Diabetes

In non-obese diabetic (NOD) mice, BTK-deficiency ameliorated disease by increasing BCR editing, thereby reducing the number of autoreactive BCRs, and so a loss in pathogenic autoantibodies. However, autoreactive B cells were still able to escape selection, and the phenotype could be restored by provision of an insulin-specific BCR (Kendall et al., 2009; Bonami et al., 2014). In another study, treatment of NOD mice with a SYK inhibitor delayed the onset and progression of the anti-insulin response (Colonna et al., 2010). These data suggest that targeting BCR signaling, and BTK in particular, could be beneficial in diabetes patients.

### Granulomatosis With Polyangiitis

In granulomatosis with polyangiitis (GPA) patients, BTK levels were increased in peripheral B cells of patients with active disease but not patients in remission, indicating its association with disease activity (von Borstel et al., 2019). Newly emerging transitional and naïve B cells were more responsive to BCR stimulation as pBTK and pPLCγ2 stimulation ratios were increased compared with healthy controls. *In vitro* incubation of patients’ B cells with acalabrutinib reduced cytokine production and plasma cell differentiation, although this reduction was smaller than in B cells from healthy controls (von Borstel et al., 2019). Nevertheless, targeting BCR signaling through BTKi could be a new treatment option in GPA.

### Pemphigus

Pemphigus and pemphigoid are AID characterized by blistering and erosions of the skin or mucosal membranes and associated with IgG autoantibodies targeting structural proteins in epithelia. Therapy involves high-dose corticosteroids and rituximab, which achieves remissions in ∼80% of patients (Bieber et al., 2021). Because of the prominent role of autoantibodies, BTKi were evaluated in canine pemphigus foliaceus and facilitated good responses (Goodale et al., 2020a, b). Efficacy of BTKi is currently evaluated in phase II (NCT02704429) and III (NCT03762265) clinical trials.

### Immune Thrombocytopenic Purpura

Immune thrombocytopenic purpura (ITP) is an AID characterized by autoantibodies targeting thrombocytes. BTKi showed effectivity in a mouse model (Langrish et al., 2017) and a phase I/II clinical trial is currently ongoing (NCT03395210) with first results indicating clinical activity (Kuter et al., 2020).

### Idiopathic Pulmonary Fibrosis

Increased BTK expression was found in circulating B cells in a fraction of patients with idiopathic pulmonary fibrosis (IPF; Heukels et al., 2019). However, BTKi showed divergent effects in bleomycin mouse models for pulmonary fibrosis, likely due to off-target effects and multi-kinase inhibition (Gu et al., 2018; Sun et al., 2020).

## BTK and BTKi Beyond the B Cell Compartment

### Psoriasis

Psoriasis is an autoinflammatory disease of the skin characterized by epidermal hyperplasia and parakeratosis. Hereby, TLR-activated myeloid cells produce cytokines critical for differentiation of IL-17 and IL-22-producing T cells. The finding that BTKi attenuated TLR7-driven psoriasis-like inflammation in mice, most likely by acting on innate immune cells (Al-Harbi et al., 2020; Nadeem et al., 2020), points to BTKi as a promising therapeutic option.

### Chronic Graft-Versus-Host Disease

Chronic graft-versus-host disease (GvHD) is a serious and life-threatening complication of allogeneic hematopoietic stem cell transplantation. Although primarily mediated by donor T cells, an important role for B cells in the disease is supported by clinical benefit of B cell depletion by rituximab. BTKi by ibrutinib could could reverse established GvHD in various T cell-driven and alloantibody-driven mouse models (Dubovsky et al., 2014; Schutt et al., 2015). A phase Ib/II clinical trial with ibrutinib in active chronic GvHD patients shows substantial clinical responses with effects on both B and T cells (Miklos et al., 2017). Based on the observed efficacy and acceptable safety, ibrutinib has been FDA-approved for treatment of GvHD patients in which prior therapy failed.

### Asthma and Chronic Obstructive Pulmonary Disease

In line with the critical role of BTK and IL-2-inducible T cell kinase (ITK) in mast cell degranulation and of ITK in T cell activation (Liao and Littman, 1995; Forssell et al., 2005), ibrutinib suppressed allergic airway inflammation in mice (Phillips et al., 2016; Nadeem et al., 2019) and blocked allergen-induced contraction of human bronchi (Dispenza et al., 2020). Furthermore, BTKi suppressed the alveolar changes related to chronic obstructive pulmonary disease (COPD) progression in mice following cigarette smoke exposure (Florence et al., 2018b), possibly by affecting airway neutrophils. Therefore, BTK/ITK inhibition in models of airway inflammation may affect both B, T, and myeloid cell activation.

### Atherosclerosis

Evidence was provided that BTKi targeting glycoprotein GPIb and GPVI signal transduction in platelets blocked atherosclerotic plaque-selective platelet aggregation but spared physiologic hemostasis (Busygina et al., 2018), implying that BTKi holds therapeutic promise in atherosclerosis.

### Coronavirus Disease 2019

Bruton’s tyrosine kinase has emerged as a potential therapeutic target to dampen the hyperinflammatory response in coronavirus disease 2019 (COVID-19). A dysregulated response by macrophages recognizing the single-stranded RNA of SARS-coronavirus-2 via TLRs is thought to be damaging to the host in severe COVID-19 disease (Merad and Martin, 2020). This is likely to involve BTK-dependent pathways including NF-κB and NLRP3 inflammasome activation, resulting in pro-inflammatory cytokine secretion.

Several lines of evidence suggest that BTKi may reduce COVID-19 symptoms. First, BTKi protected against fatal lung injury in bacterial or influenza-induced acute respiratory distress syndrome mouse models (Krupa et al., 2014; Florence et al., 2018). Second, an unexpectedly mild course of COVID-19 was seen in XLA patients (Miloševiæ et al., 2020; Quinti et al., 2020; Soresina et al., 2020) and in BTKi-treated COVID-19 patients with a B cell malignancy (Thibaud et al., 2020; Treon et al., 2020). Third, in a prospective study of hospitalized COVID-19 patients, acalabrutinib was administered off-label, and oxygenation improved, lymphopenia recovered, and inflammatory parameters normalized (Roschewski et al., 2020).

Nevertheless, very recent studies involving larger cohorts of COVID-19 patients with chronic lymphocytic leukemia indicated BTKi exerted only a modest protective effect and did not impact survival (Mato et al., 2020; Scarfò et al., 2020). A randomized phase II clinical trial of acalabrutinib in hospitalized patients was initiated (NCT04346199) but failed to meet the primary end point of increasing the proportion of patients remaining alive and free of respiratory failure. Other BTKi, including abivertinib and ibrutinib, are currently investigated in various clinical trials (NCT04528667, NCT04440007, and NCT04439006). These studies are expected to reveal whether—and at what stage of the disease—BTKi may show efficacy. Evidently, these should include the analysis of the effects of BTKi on the virus-specific antibody response and B cell memory formation.

## Conclusion and Future Perspectives

Bruton’s tyrosine kinase was discovered for its crucial role in B cell development. Ever since then, its function in very different signaling pathways and cell types has been studied in health and disease. Concordantly, multiple BTKi were developed in the context of B cell–mediated disease, especially for B cell malignancies. This spotlight is now extended toward AID, in which BTK plays an important role in pro-inflammatory activation pathways in both B and myeloid cells. This fuels further research into BTK’s exact pathogenic role in AID. These studies remain challenging because of developmental problems in BTK-deficient mice or off-target effects of BTK-inhibiting compounds, such as ibrutinib, may obscure an accurate picture of the effects of specific BTKi. Nonetheless, preliminary studies in human AID and animal models show potential clinical effectiveness of BTKi. Additionally, they not only show potential in the field of AID, but also in other diseases showing hyperinflammation, such as COVID-19, or in which autoimmunity may not be that prominent, including IPF.

Future research should aim at gaining more knowledge on the pathogenic role of BTK signaling and the effects of its inhibition in inflammatory and AID. Importantly, these disorders often involve aberrant activation of other cell types of the immune system, T cells in particular. BTK is normally not expressed in T cells,^1^ but some of the currently available BTKi have considerable off-target effects on signaling molecules expressed in T cells, including TEC, ITK, Janus kinase 3 (JAK3), or lymphocyte-specific protein tyrosine kinase (LCK; Estupiñán et al., 2021). It is attractive to explore the benefits of BTKi that show additional specificity to these related kinases as was observed in the treatment of human chronic lymphocytic leukemia or GvHD in mice (Schutt et al., 2015; Long et al., 2017). Likewise, BTK inhibition was shown to dampen inflammatory arthritis by blocking B cell activation and proliferation as well as by abolishing FcγR-induced production of pro-inflammatory cytokines in macrophages (Di Paolo et al., 2011). However, inhibitors with lower specificity also have greater off-target effects. Ibrutinib-related adverse events, including atrial fibrillation and hemorrhage, were not observed during treatment with acalabrutinib, which has improved specificity (Byrd et al., 2016). To prevent adverse events, BTKi with higher specificity are currently being developed and tested in the clinic (Estupiñán et al., 2021; von Hundelshausen and Siess, 2021). Effectiveness of strategies using combinational therapies should also be explored. In treatment of B cell malignancies, such combinational or sequential therapeutic strategies are often focused on combinations with other inhibitors acting on B cells [e.g., idelalisib targeting phosphoinositide 3-kinase (PI3K; de Rooij et al., 2015; Pal Singh et al., 2018)]. In the field of AID, however, a combination with inhibitors of T cell activation or general immunomodulatory therapies may yield high efficacy (Gillooly et al., 2017). Further studies are warranted to learn which AID patient benefits most from which therapeutic strategy.

## Author Contributions

SN, RH, and OC wrote the manuscript. All authors agreed to be accountable for the content of the work.

## Conflict of Interest

The authors declare that the research was conducted in the absence of any commercial or financial relationships that could be construed as a potential conflict of interest.

## Footnotes

1 http://rstats.immgen.org/Skyline/skyline.html
